# Highland Barley Improves DSS‐Induced Ulcerative Colitis in C57BL/6J Mice

**DOI:** 10.1002/fsn3.70132

**Published:** 2025-05-05

**Authors:** Huawei Liu, Wen Zhao, Hongzhou Chen, Hongya Wu, Xiangfei Li, Anxiang Su, Yingjian Lu

**Affiliations:** ^1^ College of Food Science and Technology Nanjing Agricultural University Nanjing China; ^2^ State Key Laboratory of Desert and Oasis Ecology, Key Laboratory of Ecological Safety and Sustainable Development in Arid Lands, Xinjiang Institute of Ecology and Geography Chinese Academy of Sciences Urumqi China; ^3^ Anhui Guo Tai Zhong Xin Testing Technology Co., Ltd Hefei China; ^4^ Lixiahe Institute of Agricultural Sciences Yangzhou China; ^5^ College of Food Science and Engineering Nanjing University of Finance and Economics/Collaborative Innovation Center for Modern Grain Circulation and Safety Nanjing China

**Keywords:** anti‐inflammatory factor, gut microbiota, highland barley, ulcerative colitis

## Abstract

The prevalence of ulcerative colitis (UC) increases with unhealthy eating habits. Both surgery and medication have the potential to treat the condition, but they may also have more negative effects. This study investigated the anti‐inflammatory mechanism of 20% and 40% doses of different highland barley (HB) components (whole grain, peeled, and bran) in a 2% dextran sulfate sodium induced UC mouse model. The results showed that supplementation with a 20% dose of peeled HB restored body weight, disease activity index, colon length, serum interleukin‐1β and interleukin‐10 levels, liver glutathione peroxidase content, and superoxide dismutase activity to normal levels in mice compared to UC mice. Moreover, the damage caused by UC to the mice's colon was significantly reduced, and the relative expression levels of interleukin‐1β, interleukin‐6, and tumor necrosis factor‐α were all significantly downregulated. Additionally, it increased the abundance of *Bacteroidota* and *Firmicutes*, improving the balance of gut microbiota.

## Graphical abstract

Supplementation with a 20% dose of peeled highland barley restored body weight, disease activity index, colon length, serum IL‐1β and IL‐10 levels, liver GSH‐Px content, and SOD activity to normal levels in mice compared to ulcerative colitis mice. The damage caused by ulcerative colitis to the mice's colon was significantly reduced, and the relative expression levels of IL‐1β, IL‐6, and TNF‐α were all significantly downregulated. Additionally, it increased the abundance of *Bacteroidota* and *Firmicutes*, improving the balance of gut microbiota.

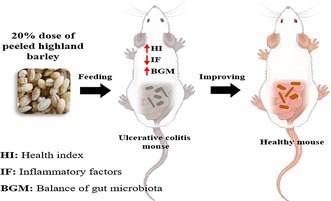

**Abbreviations**
BC: blank control
BHT: high‐dose peeled HB
BLT: low‐dose peeled HB
DAI: disease activity index
DSS: dextran sulfate sodium
EM: emulation modeling
FOBT: fecal occult blood test
GSH‐Px: glutathione peroxidase
HB: highland barley
HBB: high‐dose HB bran
HWG: high‐dose HB whole grain
IFN‐γ: interferon‐γ
IL‐10: interleukin‐10
IL‐1β: interleukin‐1β
IL‐6: interleukin‐6
LBB: low‐dose HB bran
LWG: low‐dose HB whole grain
MDA: malondialdehyde
OTU: operational taxonomic units
PC: positive control
SCFAs: short‐chain fatty acids
SOD: superoxide dismutase
TNF‐α: tumor necrosis factor‐α
UC: ulcerative colitis

## 1 Introduction

Ulcerative colitis (UC) is a global but distinctly geographic chronic gastrointestinal disease whose incidence has increased dramatically over the past 50 years (Roberts et al. 2020), especially in newly industrialized countries or in adolescent groups; the diet structure has changed dramatically, caused by fast food, accelerating the emergence of various gastrointestinal diseases (Corsello et al. 2020; Thomas et al. 2020; Gilaad and Siew 2016). Although the etiology of UC is unknown, many factors, including genetic factors, external environment, dietary habits, abdominal surgery, and immune causes, increase the prevalence of UC (Kobayashi et al. 2020). In some patients, it mostly causes weight loss, stomach pain, and loose stool. Therefore, UC not only brings a heavy medical burden to the Western developed countries (Coward et al. 2019), but also seriously drags down the economic development of South America, Eastern Europe, Asia, Africa, and other regions and brings a great challenge to the global health care system (Ng et al. 2018).

At present, surgery and medication are the main approaches to therapy for UC, and surgical therapy is mainly to remove the diseased part of the patient's colon to prevent its further spread. However, surgery will reduce the quality of life of patients and increase the risk of cancer in patients (Ocansey et al. 2020), and the mortality rate of patients after surgery is 5.5%. Therefore, the pharmacological treatment methods, including aminosalicylic acid preparation therapy, immunosuppressive therapy, anti‐inflammatory drug therapy, and glucocorticoid therapy, were usually used to relieve UC through enhancing the patient's immunity, anti‐inflammatory properties, and repair of intestinal mucosa, and so forth. However, these treatments do not completely cure UC and may cause recurrence of the disease, along with numerous adverse effects. A growing number of studies have demonstrated the potential of some natural cereal food components to prevent and alleviate the symptoms of UC. Bamba et al. (2002) found that germinated barley foodstuff was effective in treating UC by modulating gut microbiota. Supplementation of wheat bran polyphenols inhibited the MAPK/NF‐κB inflammasome pathway, improved colon morphology, and intestinal microbiota in UC mice (Wen et al. 2024). Liu et al. demonstrated that a quinoa whole grain diet was effective in reducing disease activity index and histological damage in mice, ameliorating UC‐induced intestinal dysbiosis, and reducing overgrowth of the *Proteobacteria* phylum (Liu et al. 2018). However, there are no studies on the effects of highland barley (HB) on UC.

HB, also known as naked barley, is a variety of barley of the genus *Erigerus* of the family *Gramineae* and is one of the main food crops on the Tibetan plateau. It is a very important plateau cereal crop planted in high‐altitude and cold zones, with characteristics of barren resistance, cold resistance, short growth period, high yield, early maturity, and wide adaptability (Lyu et al. 2022). In addition, HB is rich in plenty of functional components, including glucans, flavonoids, polyphenols, dietary fiber, and other active substances. Compared to other cereals, the unique composition of HB has enabled it to show higher efficacy in anti‐inflammatory, anticancer, hypoglycemic, antimicrobial, anti‐obesity, anti‐fatigue, anti‐aging, hypoglycemic, and hypolipidemic properties (Obadi et al. 2021). Although HB has been used as food and feed crops for a long time, the problems of single use and low added value have become increasingly prominent. Chen et al. (2019) found that β‐glucan isolated from HB increased the length, faces water contents, and total short‐chain fatty acids (SCFAs) concentration of the mice colon and cecum, showing a protective effect on the intestinal tract. On the other hand, studies have shown that dietary fiber intake plays an active role in the prevention of inflammatory bowel disease by enhancing the diversity of the gut microbiota and modulating its effects on the mucosal immune system (Gentschew and Ferguson 2012). The purpose of the current study was to explore the protective effect of HB supplementation on the improvement of UC, to explore the potential application value of HB, and to provide a basis for further utilization and rational development of HB. Therefore, we hypothesized that HB supplementation could ameliorate DSS‐induced UC in mice and that this effect was related to gut microbiota. In the present study, we investigated the relationship between the levels of relevant inflammatory factors, antioxidant indices, tissue sections, and gene transcripts of tissues and gut microbiota with HB whole grain, peeled HB, and HB bran. This study will provide new insights for future UC prevention and development of functional HB foods.

## 2 Methods and Materials

### 2.1 Materials and Reagents

The customized HB whole grain, peeled HB, and HB bran were prepared by Zhongwang Food Co. Ltd. (Jiangsu, China). The starch content determination kit was purchased from Beijing Solarbio Science and Technology Co. Ltd. (Beijing China). The mixed‐linkage beta‐glucan assay procedure was afforded by Megazyme International Ireland (Ireland). Dextran sulfate sodium (DSS, molecular weight 36,000–50,000) was obtained from MP Biomedicals LLC (Solon, OH, USA). The fecal occult blood test (FOBT) kit (o‐toluidine method) was supplied by Shanghai yuanye Bio‐Technology Co. Ltd. (Shanghai, China). Glutathione peroxidase (GSH‐Px), MPO, superoxide dismutase (SOD), and malondialdehyde (MDA) assay kit was offered by Jiancheng Bioengineering Institute (Nanjing, China). The ELISA Kit for interleukin‐1β (IL‐1β), interleukin‐6 (IL‐6), interleukin‐10 (IL‐10), tumor necrosis factor‐α (TNF‐α), and interferon‐γ (IFN‐γ) was bought from Elabscience Biotechnology Co. Ltd. FastPure Cell/Tissue Total RNA Isolation Kit V2, HiScript III All‐in‐one RT SuperMix Perfect for qPCR, and Taq Pro Universal SYBR qPCR Master Mix was provided by Vazyme Biotech Co. Ltd. (Nanjing, China). Other chemicals and analytical reagents were obtained from Nanjing Chemical Reagents Co. Ltd. (Nanjing, China).

### 2.2 Determination of Components of Highland Barley

The starch content determination kit was used to determine the starch content in HB whole grain, peeled HB, and HB bran, strictly in accordance with the kit instructions. The β‐glucan content in HB whole grain, peeled HB, and HB bran was determined using the β‐glucan quantification kit, which was operated strictly in accordance with the kit instructions. The moisture content in HB whole grain, peeled HB, and HB bran was determined by using a rapid moisture tester (BLH‐103MW, Zhejiang Bethlehem Instruments & Equipment Co. Ltd.) and measured three times in parallel.

Flavonoid and total phenol content was determined using the method of Mao et al. (2020). The HB whole grain, peeled HB, and HB bran were crushed, and the dried powder and 80% ethanol were mixed in the ratio of 1:10 by mass to volume, respectively, and then the mixture was extracted by ultrasonic extraction for 1 h and centrifuged at 5000 rpm for 10 min, and the supernatant was taken as the prepared sample solution. Then, 0.5 mL of the above prepared sample solution was pipetted into a clean centrifuge tube, and 2 mL of double‐distilled water and 0.15 mL of 10% NaNO_2_ solution were added sequentially, vortexed, and mixed well. After 5 min, 0.15 mL of 10% AlCl_3_ solution was added, and the solution was mixed with upside‐down shaking and left for 5 min. Then, 1 mL of 1 mol/L NaOH solution was added and mixed thoroughly and left for 15 min. The absorbance was measured by UV spectrophotometer at 415 nm. The standard curve was prepared using rutin as the standard, and the flavonoid contents in HB whole grain, peeled HB, and HB bran were calculated. Another 0.5 mL of the prepared sample solution was taken as described above in a clean centrifuge tube, and 2 mL of 7.5% NaCO_3_ solution and 2.5 mL of Folin–Ciocalteu reagent were added in turn, mixed thoroughly, and left to react for 2 h at room temperature while avoiding light. The absorbance of the mixture was measured at 765 nm by UV spectrophotometer, and the total phenol content was expressed as gallic acid equivalent.

### 2.3 Animal Experimental Design

All animal experimental protocols used in this research were conducted in strict accordance with the Ethical Standards for Laboratory Animal Welfare (DB32/T‐2911‐2016) and approved by the Institutional Animal Care and Use Committee of Nanjing Agricultural University (SYXK‐2017‐0027). A total of 72 specific pathogen‐free (SPF) 8‐week‐old male C57BL/6J mice (19–21 g) were purchased from Shanghai Slac Laboratory Animal Co. Ltd. (Shanghai, China). They were regularly fed while being kept in a 20°C–22°C, 12 h light/dark cycle environment.

**FIGURE 1:**
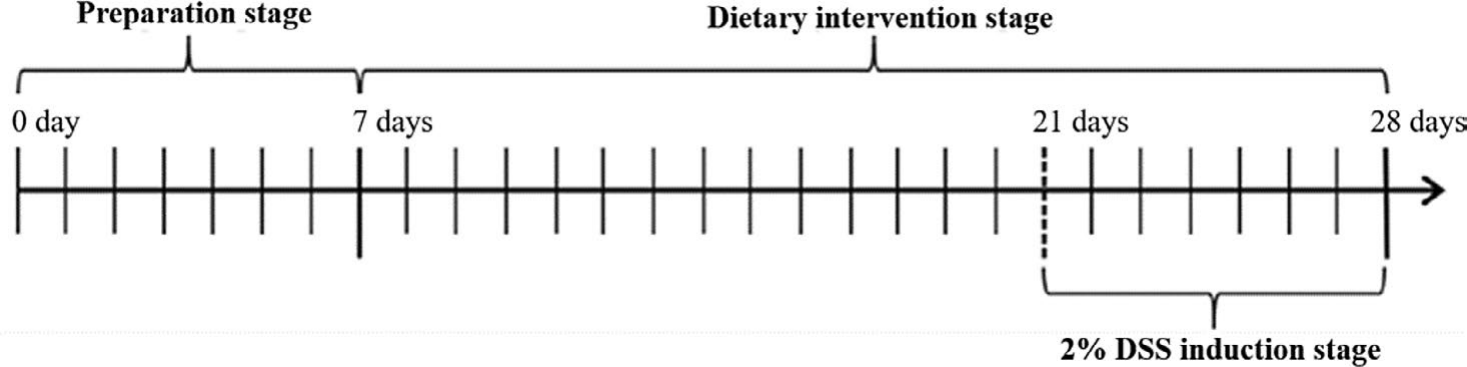
Schematic diagram of the modeling cycle. 0–7 days, preparation stage. 7–21 days, dietary intervention stage. 21–28 days, 2% DSS induction stage.

After 1‐week adaptation, all 72 mice were randomly divided into nine groups (*n* = 8), which were named as blank control (BC) group, emulation modeling (EM) group, positive control (PC) group, low‐dose HB whole grain (LWG) group, high‐dose HB whole grain (HWG) group, low‐dose peeled HB (BLT) group, high‐dose peeled HB (BHT) group, low‐dose HB bran (LBB) group, and high‐dose HB bran (HBB) group. The schematic diagram of mouse modeling is shown in Figure 1. For the first 14 days, the BC, EM, and PC groups were fed normal feed, and the remaining groups were fed the corresponding customized feed. For the last 7 days, normal drinking water was replaced with 2% DSS solution in all groups except the BC group. The PC group was gavaged with 0.4 mL of 3% salicylate sulfapyridine solution, and the remaining groups were gavaged with the same amount of water (Yan et al. 2019). During the experiment, the body weights of all mice were measured every 3 days for the first 14 days and daily for the last 7 days. Fecal occult blood tests were performed on mice feces every 3 days during the last 7 days of modeling, strictly following the kit procedure. After 21 days, all mice were fasted for 12 h and sacrificed with cervical vertebrae dislocated after collecting blood samples. The blood samples were centrifuged at 3000 × *g* at 4°C for 15 min to extract serum, and the serum samples were collected in sterile and enzyme‐free centrifuge tubes. The colon length of mice was measured by vernier calipers and recorded. The serum, liver, spleen, kidneys, colon, colon contents, small intestine, cecum, and cecum contents were taken and stored at −80°C for the following experiments.

### 2.4 Evaluation of Disease Activity Index

In the last 7 days of modeling, mouse feces were collected on a white porcelain plate every 3 days, and the degree of fecal occult blood was determined by using a qualitative FOBT kit, which was used as a scoring criterion for the disease activity index (DAI) score. The FOBT kit used the o‐toluidine method. The results of the fecal occult blood test were interpreted according to the criteria shown in Table 1 and scored on the basis of these criteria as detailed in Table 2. The scores of each indicator are summed to obtain the DAI index.

**TABLE 1:** Standard for interpretation of fecal occult blood test results.

Color change	Score
Blue‐black‐brown immediately after adding reagent	4+
Blue‐brown immediately after adding reagent, and gradually dark brown	3+
Light green at the beginning after adding reagent, and gradually become obvious blue‐brown	2+
Gradual change from light green to green after 10 s	+
No color development within 2 min	0

**TABLE 2:** DAI index scoring criteria.

Score	Rate of weight loss	Stool consistency	Level of fecal occult blood
0	No change or increase in body mass	Normal stool	Negative occult blood test
1	Weight loss of 1%–5%	Soft stool, globular stool	Weakly positive occult blood test
2	Weight loss of 5%–10%	Loose paste or hemispherical stool, no anal attachment	Positive occult blood test
3	Weight loss of 10%–15%	Paste‐like stool with anal attachment	Strongly positive occult blood test
4	Weight loss of more than 15%	Diarrhea, loose stool or watery stool	Blood in the stool or anus

### 2.5 Histopathological Analysis

The 1 cm middle section of mouse colon tissue was placed in a sterile EP tube, submerged in 4% paraformaldehyde, and fixed for 24 h. It was then transferred to Shanghai Irvine Biotechnology Co. Ltd. for fixation, pathological observation by H&E staining, and PAS staining, followed by laboratory light microscopy photography for pathological examination.

### 2.6 Detection of Inflammatory Factor Levels

Levels of IL‐1β, IL‐10, IL‐6, and other markers in the serum described above were determined in strict accordance with the kit instructions.

### 2.7 Determination of Liver Antioxidation Index

The 0.1 g liver tissue was added to nine times the volume of sterile saline, homogenized thoroughly using a tissue grinder, and then the supernatant was centrifuged at 10,000 rpm for 10 min at 4°C in a sterile centrifuge tube. The contents of SOD and GSH‐Px in the supernatant of liver homogenate were determined by strictly observing the requirements of the kit instructions.

### 2.8 Real‐Time PCR Analysis

RNA extraction and determination of gene expression were performed using the method of Tang et al. (2021) with some modifications. The total RNA was extracted from the appropriate colonic tissue using the tissue RNA rapid extraction kit, and then transcribed into cDNA using the reverse transcription kit. The cDNA was subjected to fluorescent quantitative PCR using SYBR Green dye to determine the mRNA expression level of each gene. Table 3 displays a list of qPCR primers, and the fluorescent quantitative PCR reaction system is shown in Table 4. The following steps were used in the RT‐PCR reaction: 50°C for 2 min, 95°C for 5 min, 95°C for 15 s, 60°C for 1 min, 95°C for 15 s, 60°C for 1 min, 95°C for 15 s, 40 cycles. The relative expression levels of target genes were calculated by the $2^{-\Delta \Delta C_{t}}$ method.

**TABLE 3:** The sequences were detected by real‐time PCR primers.

Gene	Primer sequences (5′–3′)
β‐actin	F: GTTACCACTGGGACGAC
	R: CTCAAACATGATCTGGGTCA
TNF‐α	F: TCTTCTCATTCCTGCTTGTGG
	R: GGTCTGGGGCATAGAACTGA
IL‐1β	F: AACCTGCTGGTGTGTGACGTTC
	R: CAGCACGAGGCTTTTTTGTTGT
IL‐6	F: TACCACTTCACAAGTCGGAGGC
	R: CTGCAAGTGCATCATCGTTGTTC

**TABLE 4:** The composition of real‐time PCR system.

Reagents	Dosage
cDNA template	2 μL
Upstream primers	0.8 μL
Downstream primers	0.8 μL
dd H_2_O	6.4 μL
SYBR Green	10 μL

### 2.9 Analysis of Gut Microbiota Composition

The interactive microbial community diversity, cloud analysis, and bioinformatics analysis were conducted to investigate the microbiota of mouse feces, which were collected in sterile EP tubes and quickly frozen in liquid nitrogen, and then shipped to Shanghai Meiji Biological Company on dry ice for sequencing. The obtained reads from sequencing were first spliced according to the overlap relationship, and then quality control and filtering were carried out to distinguish the samples for operational taxonomic units (OTU) clustering analysis and species taxonomy.

### 2.10 Statistical Analysis

All experimental data were analyzed and processed using IBM SPSS 26.0 software, which utilized one‐way analysis of variance (ANOVA) and Tukey's test; the difference was regarded as analytically significant if *p* < 0.05. All results were expressed as mean ± standard error of the mean (SEM).

## 3 Results

### 3.1 Composition of HB

Notably, both starch and β‐Glucan contents decreased significantly in peeled black barley, HB grain, and barley bran in turn (*p* < 0.05). Table 5 displays the findings of measuring the fundamental components of HB whole grain, HB bran, and peeled HB. The moisture content of the three types of barley was highest in the HB whole grain, significantly greater than in the HB bran and peeled HB, and the HB bran significantly higher than the peeled HB (*p* < 0.05). The highest flavonoid and total phenol concentration was found in HB bran, which was considerably greater than both peeled HB and HB whole grain (*p* < 0.05). Nevertheless, HB whole grain had a significantly higher flavonoid and total phenol content than peeled HB (*p* < 0.05). Notably, both starch and β‐glucan contents decreased significantly in peeled black barley, HB grain, and barley bran in turn (*p* < 0.05).

**TABLE 5:** Basic components of HB whole grain, HB bran, and peeled HB.

Samples	HB whole grain	HB bran	Peeled HB
Starch content (%)	36.48 ± 2.08^b^	26.69 ± 0.03^c^	49.38 ± 0.97^a^
β‐Glucan content (%)	5.05 ± 0.02^b^	4.45 ± 0.06^c^	6.62 ± 0.07^a^
Moisture content (%)	12.16 ± 0.01^a^	11.49 ± 0.01^b^	10.35 ± 0.01^c^
Flavonoid content (%)	0.19 ± 0.08^b^	0.29 ± 0.08^a^	0.15 ± 0.05^c^
Total phenol content (%)	0.10 ± 0.005^b^	0.15 ± 0.003^a^	0.06 ± 0.002^c^

“a, b, c” represents significant differences between each other at the 5% level.

### 3.2 Evaluation of Weight, DAI, Colon Length, and Organ Index

**FIGURE 2:**
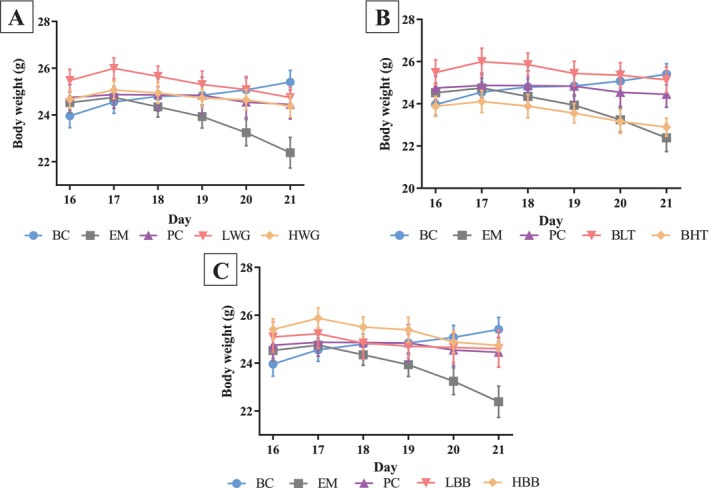
Effects of highland barley intervention on body weight of mice. (A) LWG and HWG compared with BC, EM, and PC groups. (B) BLT and BHT compared with BC, EM, and PC groups. (C) LBB and HBB compared with BC, EM, and PC groups.

As shown in Figure 2, during the 7 days of creating the UC model, the body weights of the mice in all groups except the BC group decreased to different degrees (*p* > 0.05). On the last day of modeling, the body weight of mice in the EM group was significantly lower than that of mice in the BC group (*p* < 0.05). However, compared with the BC group, high and low doses of HB whole grain, HB bran, and low doses of peeled HB significantly inhibited the weight loss of mice with UC at the end of modeling, while high doses of peeled HB did not significantly inhibit the weight loss of mice (*p* < 0.05).

**FIGURE 3:**
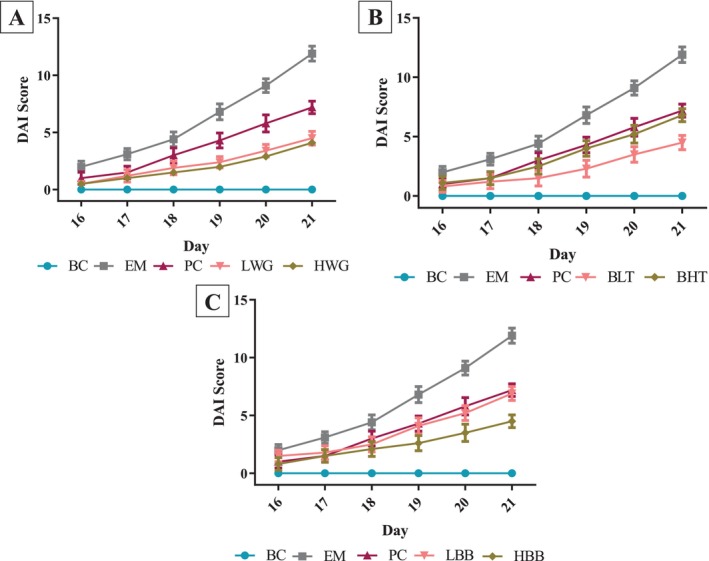
HB treatments affect the DAI in mice. (A) LWG and HWG compared with BC, EM, and PC groups. (B) BLT and BHT compared with BC, EM, and PC groups. (C) LBB and HBB compared with BC, EM, and PC groups.

Figure 3's DAI indices of body weight loss rate, fecal occult blood, and fecal morphology demonstrate that there was no discernible difference between the treatment groups during the first 3 days of simulation. From the sixth day of modeling, mice in the EM group showed blood in the stool, blood staining in the anus, and a significant decrease in mobility, as well as a significant increase in DAI compared with the BC group, indicating that the DSS reagent caused damage to the colon of the mice (*p* < 0.05). However, mice in the other groups, except for the EM group, did not show blood in the stool and decreased mobility, and the DAI was significantly lower than that of the EM group (*p* < 0.05). In addition, there was no significant difference in DAI between mice in the LWG and HWG groups (*p* > 0.05). The DAI of mice in the BLT group was significantly lower than that of mice in the BHT group, and the DAI of mice in the HBB group was significantly lower than that of mice in the LBB group (*p* < 0.05).

The colon length and organ index were measured in each group of mice (Table 6). The colon length of mice in the EM group was significantly lower than that in the BC group (*p* < 0.05), suggesting that UC shortened the colon of mice. However, supplementation with 3% salicylate sulfapyridine solution, HB whole grain, peeled HB, and HB bran significantly increased colon length compared to the EM group (*p* < 0.05). The organ index is the weight of the organ in the organism, which can further reflect the health of the organism. There was no significant difference in the organ index of each group of mice (*p* > 0.05), indicating that UC only had a significant effect on the colon tissue but not on other organs.

**TABLE 6:** Mouse colon length and organ index.

Group	Colon length (cm)	Liver/Weight (g/100 g)	Spleen/Weight (g/100 g)	Kidney/Weight (g/100 g)
BC	7.79 ± 0.58^a^	3.77 ± 0.06^a^	0.25 ± 0.02^a^	1.23 ± 0.04^a^
EM	6.12 ± 0.48^b^	3.89 ± 0.06^a^	0.29 ± 0.01^a^	1.22 ± 0.02^a^
PC	7.78 ± 0.91^a^	3.85 ± 0.09^a^	0.33 ± 0.06^a^	1.17 ± 0.03^a^
LWG	6.98 ± 0.40^a^	3.93 ± 0.10^a^	0.30 ± 0.04^a^	1.20 ± 0.03^a^
HWG	7.67 ± 0.56^a^	3.94 ± 0.07^a^	0.31 ± 0.03^a^	1.16 ± 0.03^a^
BLT	7.38 ± 0.45^a^	3.94 ± 0.11^a^	0.32 ± 0.03^a^	1.28 ± 0.04^a^
BHT	7.10 ± 0.80^a^	3.88 ± 0.13^a^	0.31 ± 0.04^a^	1.20 ± 0.05^a^
LBB	7.27 ± 0.68^a^	4.01 ± 0.11^a^	0.34 ± 0.04^a^	1.23 ± 0.03^a^
HBB	7.25 ± 0.49^a^	4.04 ± 0.06^a^	0.29 ± 0.03^a^	1.18 ± 0.02^a^

Abbreviations: BC, blank control; BHT, high‐dose peeled HB; BLT, low‐dose peeled HB; EM, emulation modeling group; HBB, high‐dose HB bran; HWG, high‐dose HB whole grain; LBB, low‐dose HB bran; LWG, low‐dose HB whole grain; PC, positive control.

“a, b, c” represent significant differences between each other at the 5% level.

### 3.3 Histopathological Analysis

**FIGURE 4:**
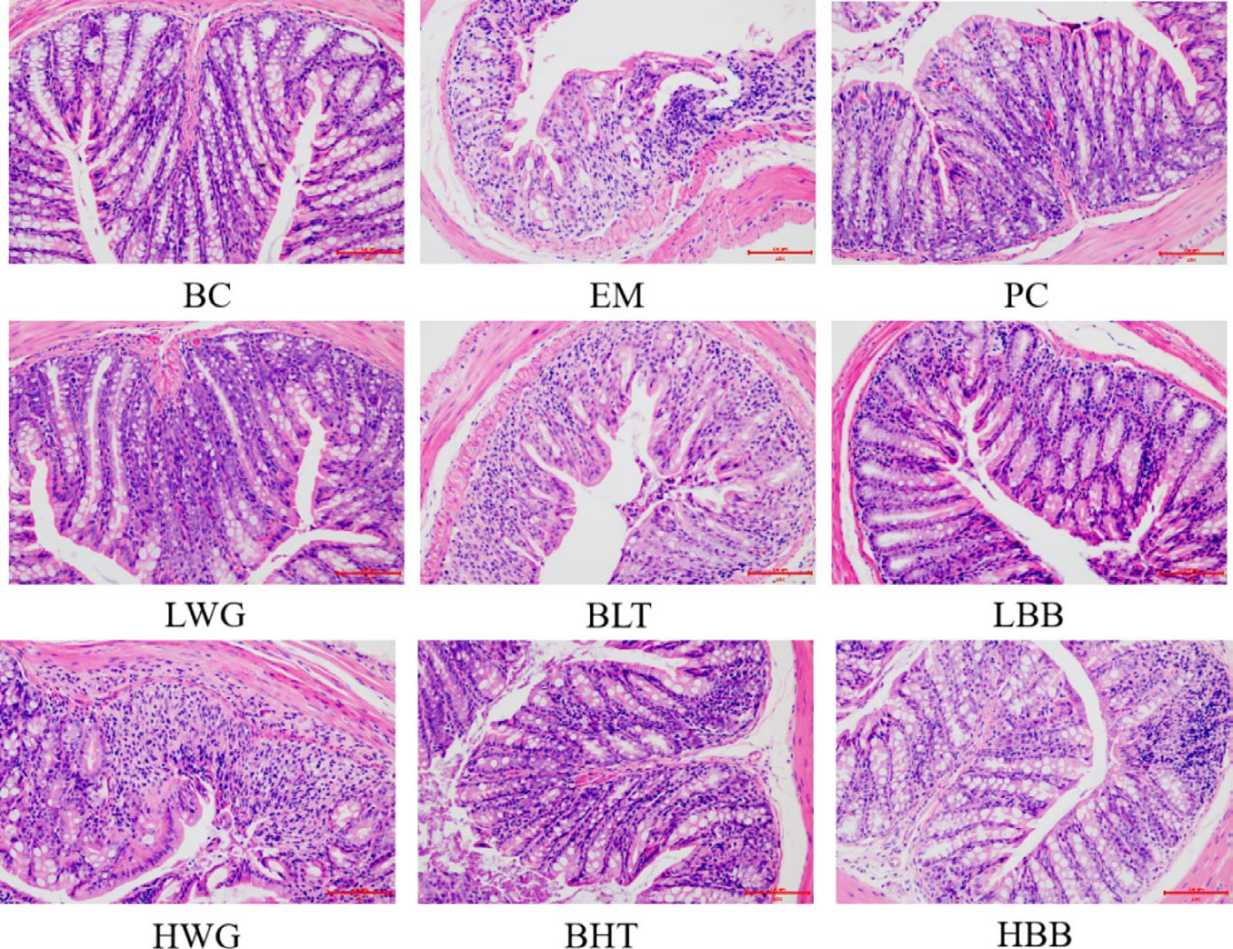
The difference in mouse colon sections caused by HB (HE staining, ×200). The groups of LWG, HWG, BLT, BHT, LBB, and HBB are compared with the BC, EM, and PC groups.

The BC group of mice had intact colonic tissue structure, intact villi structure, dense and neat crypt structure, intact glandular structure, intact and uniform size of cup‐shaped cells, and no inflammatory cell infiltration (Figure 4). Compared with the BC mice, the EM mice had disordered colonic sections, separated muscle layers, lost crypt structure, had a significant decrease in cupped cells, and had a significant infiltration of inflammatory cells. When compared to the EM mice, the LWG and LBB were able to reduce the extent of damage to mouse colon tissue, but inflammatory cells remained infiltrated, and the cup‐shaped cell structure was still unfinished. The mouse's colonic tissue tended to be intact after HWG and HBB were administered, the glandular structure was restored, the amount of inflammatory cell infiltration was decreased, the cup‐shaped cell sizes were normal, and the crypt structure was tidy and dense. Both BHT and BLT resulted in a small amount of inflammatory cell infiltration, but the BLT restored the mouse's colon tissue with no discernible difference from the BC mice. Moreover, the BLT did better than BHT. In conclusion, HWG and HBB, along with BLT, greatly reduced the UC‐related damage to mouse colonic tissues.

**FIGURE 5:**
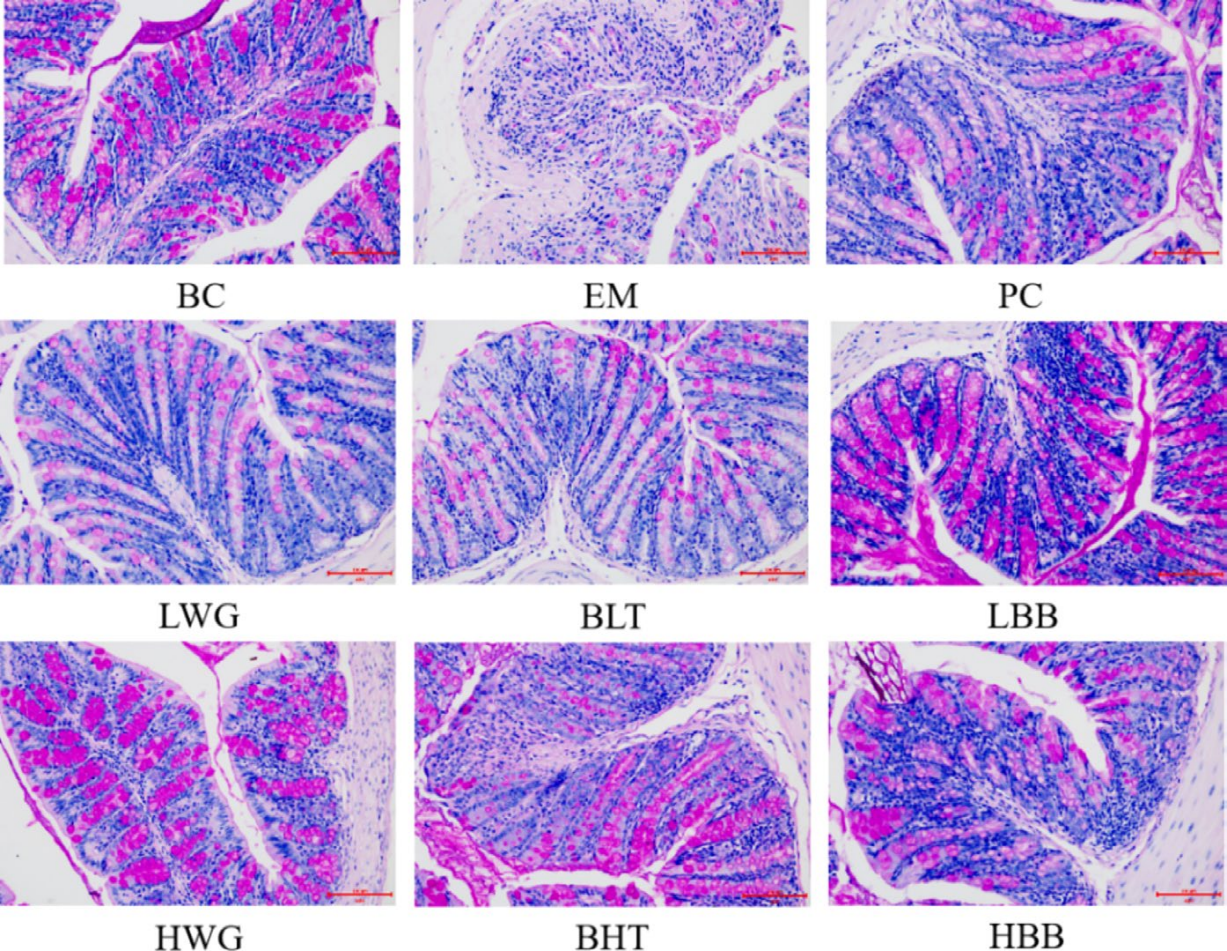
PAS staining of mice colon sections displays the difference between the HB treatments (×200). The groups of LWG, HWG, BLT, BHT, LBB, and HBB are compared with the BC, EM, and PC groups.

As shown in Figure 5, when compared to the BC mice, the colonic tissue of mice in the EM was severely damaged, with a large reduction or even loss of cup cells, loss of glandular structures, and incomplete tissue structure. In contrast, the colonic tissue of the mice in the PC group was intact, with the appearance of glandular structures and the restoration of cup cells to a state that was not significantly different from that of the BC mice, indicating that the positive drug intervention could improve the colonic damage in mice with UC. The loss of glandular structure and reduction of cup cells brought on by UC in mice resulted in all of the indexes being greatly reduced by HB whole grain, HB bran, and peeled HB, at high and low doses. In particular, the HWG, HBB, and BLT performed better. Thus, the number of cup‐shaped cells in the colon tissue of the HB intervention group was restored to the normal level, and the size of the cup‐shaped cells was uniform.

### 3.4 Evaluation of Inflammatory Factors Levels

**FIGURE 6:**
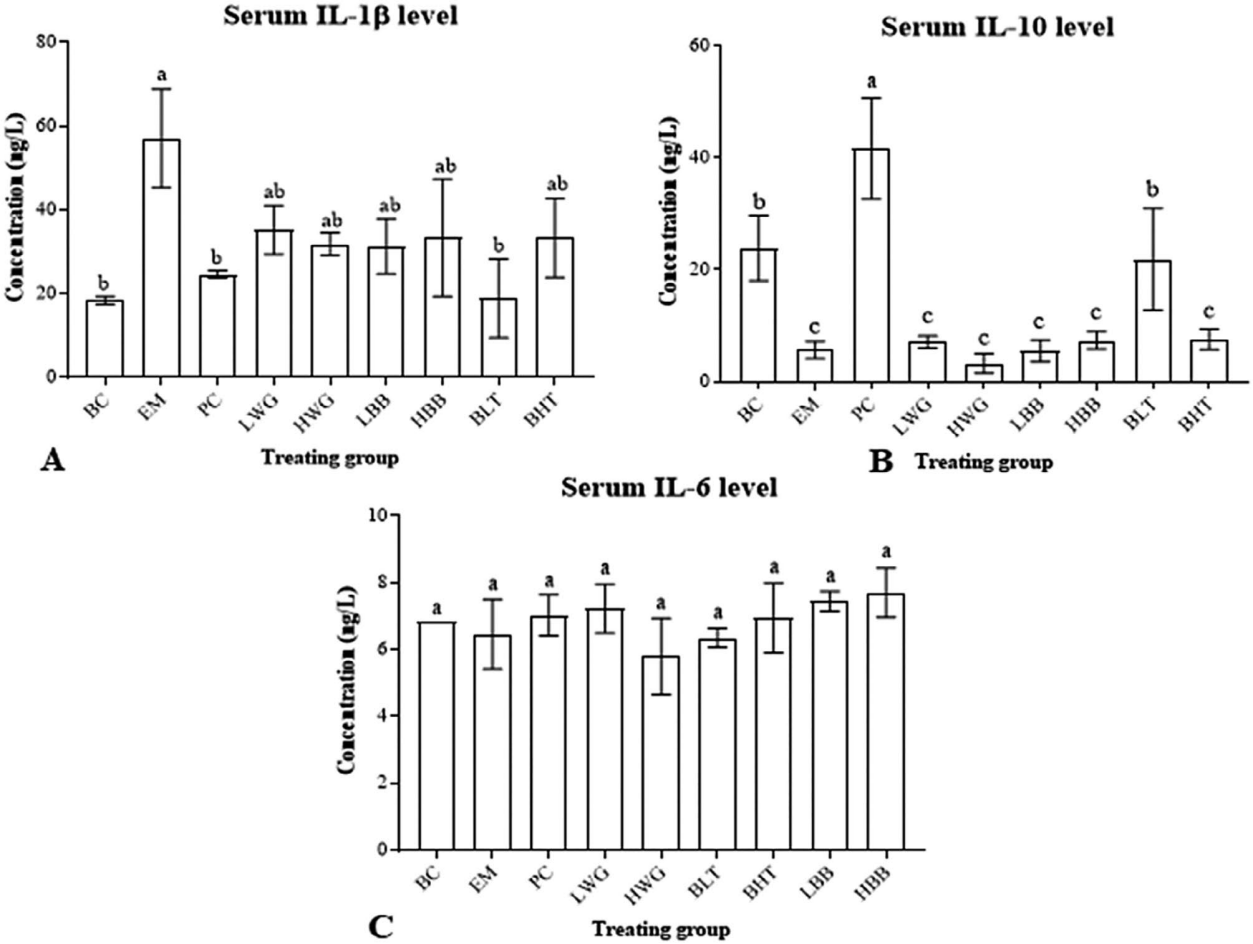
Evaluation of inflammatory factor levels. (A–C) The level of serum IL‐1β, IL‐10, and IL‐6 in BC, EM, PC, LWG, HWG, LBB, HBB, BLT, and BHT groups, separately. “a, b, c” represent significant differences between each other at the 5% level.

The pro‐inflammatory cytokine IL‐1β is mostly produced by monocytes, and UC has been linked to an increase in IL‐1β level in the body (Okada et al. 2021). Compared with the BC group, the IL‐1β levels of serum in the EM group of mice significantly increased (Figure 6A, *p* < 0.05). In the HB sample group, only the BLT group of mice significantly decreased IL‐1β levels in serum, the same as the PC mice (*p* < 0.05). The serum IL‐10 level of mice in the EM group was significantly lower than those in the BC group, but there was a significant increase in the PC group, even more than in the BC group (Figure 6B, *p* < 0.05). In those conducted HB groups, only the BLT significantly increased serum IL‐10 level, which was the same as in the BC group (*p* < 0.05). Notably, for the IL‐6 levels of serum, there was no significant difference between the BC mice, EM mice, PC mice, and HB mice (Figure 6C, *p* > 0.05).

### 3.5 Antioxidation Index Analysis

**FIGURE 7:**
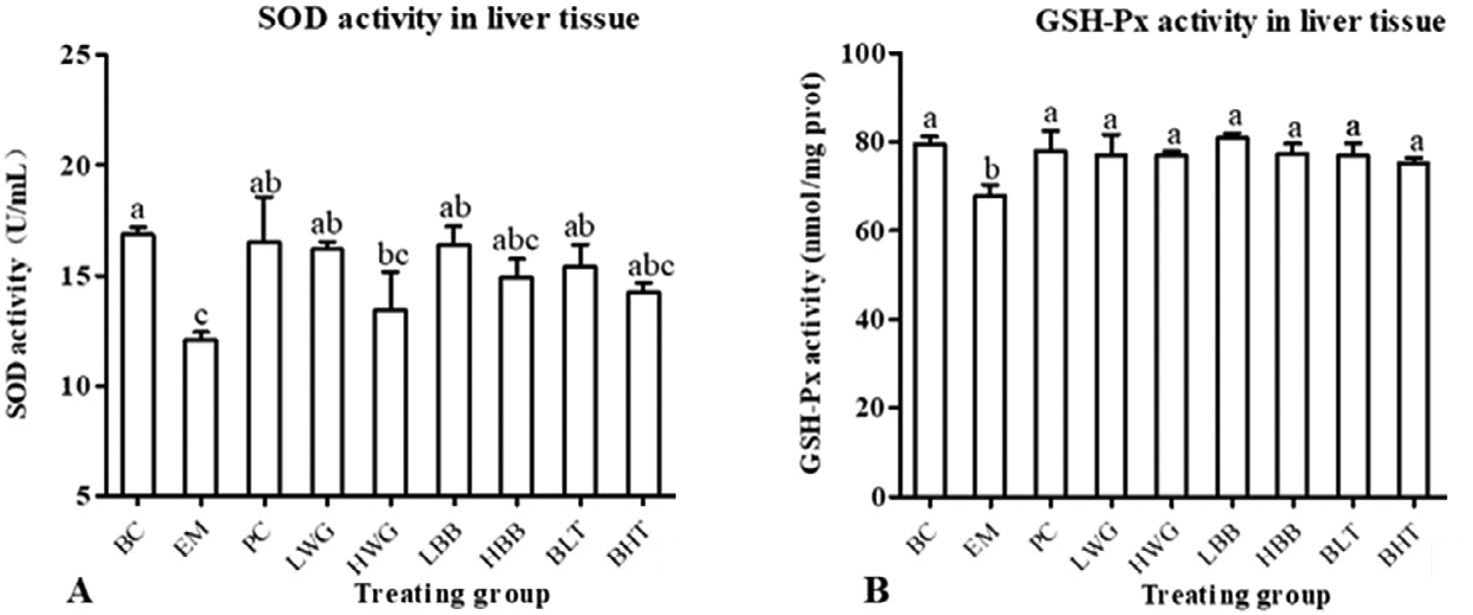
Comparison of the difference GSH‐Px and SOD in various groups. (A, B) The GSH‐Px and SOD contents in the liver of mice from different groups, including BC, EM, PC, LWG, HWG, BLT, BHT, LBB, and HBB. “a, b, c” represent significant differences between each other at the 5% level.

Compared with the BC group, SOD levels in the livers of mice in the EM group were significantly decreased (Figure 7A, *p* < 0.05). However, compared with the EM group, SOD levels in the livers of mice in the PC, LWG, LBB, and BLT groups were significantly increased (*p* < 0.05), suggesting that they could attenuate the oxidative damage in the liver caused by UC. Figure 7B showed that the GSH‐Px levels in the livers of mice in the EM group were significantly lower than those of mice in the BC group, whereas supplementation with each of the HB samples significantly increased the GSH‐Px levels in the livers of mice (*p* < 0.05). This indicated that all HB samples could alleviate the oxidative stress induced by UC by restoring hepatic GSH‐Px levels.

### 3.6 Real‐Time PCR Analysis

**FIGURE 8:**
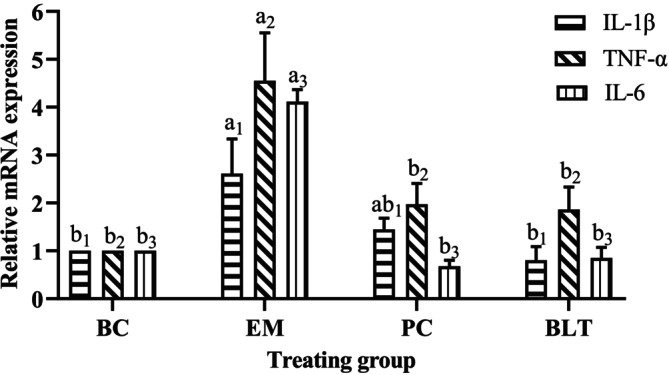
The relative expression of IL‐1β, IL‐6, and TNF‐α genes in mouse colon. “a, b, c” represent significant differences between each other at the 5% level.

To investigate the effect of HB intervention on the expression of inflammatory factor‐related genes in mice colon tissues, the gene expression of IL‐1β, IL‐6, and TNF‐α in colon tissues was determined (Figure 8). Compared with the BC group, the levels of IL‐1β, IL‐6, and TNF‐α in the colon of mice in the EM group were significantly increased, indicating that UC caused colonic inflammation (*p* < 0.05). When compared with the EM group, BLT intervention could significantly downregulate the expression of IL‐1β, IL‐6, and TNF‐α in the colon of mice (*p* < 0.05), which had a preventive effect on the inflammatory response of UC mice, and its downregulation of IL‐1β expression was better than that of the PC group.

### 3.7 Gut Microbiota Analysis

**FIGURE 9:**
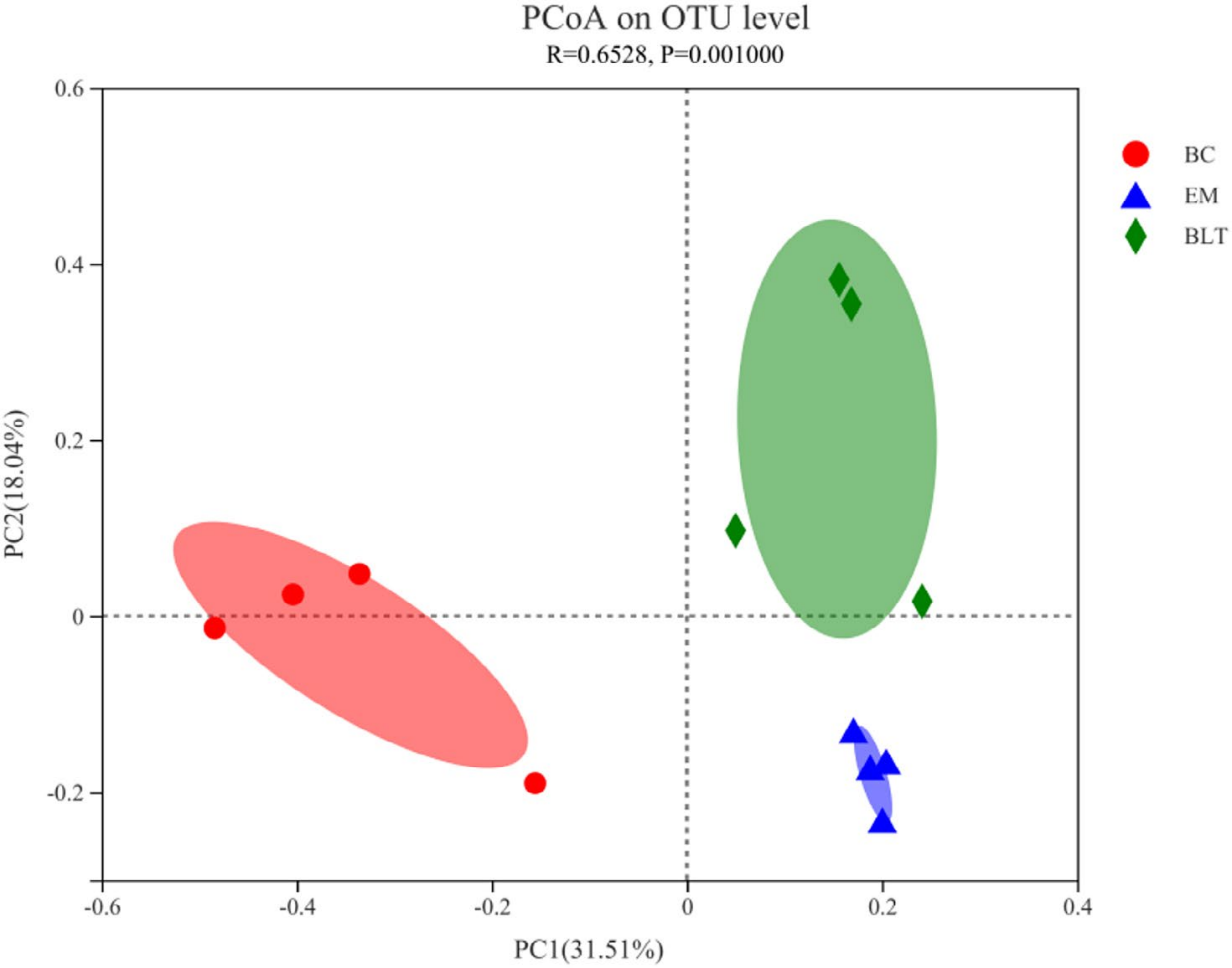
Analysis of beta diversity of intestinal microbiota in mice.

**FIGURE 10:**
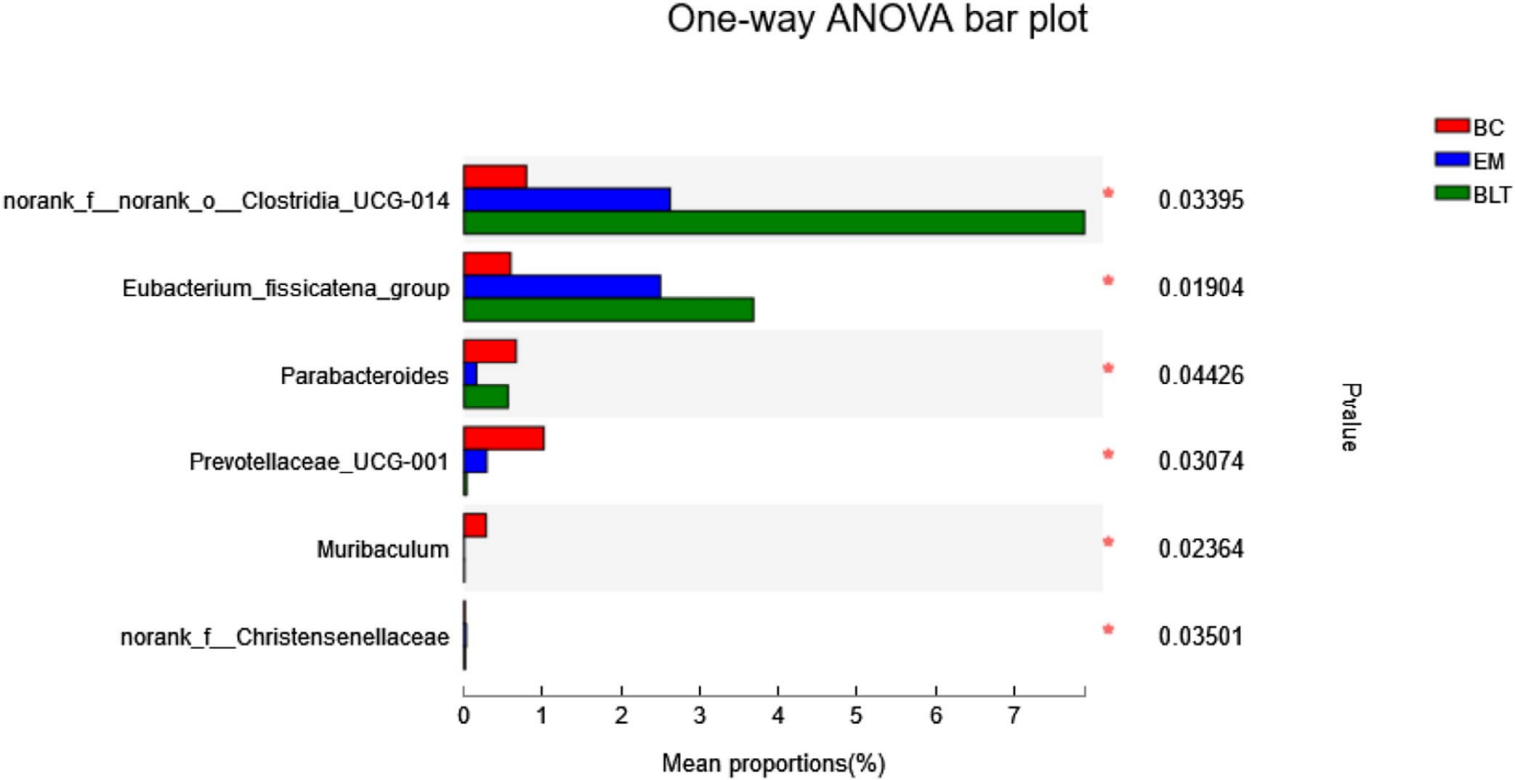
Multispecies difference test of gut microbes (genus level).

The Bray–Curtis distance method and the ANOSIM intergroup difference test were used for the analysis of the beta diversity of gut microbiota to detect the similarities and differences in species composition between various samples. The categorization level employed was the OTU level. As a result, the species composition of the BLT did not overlap with that of the BC (Figure 9). The BLT is a food‐derived substance that plays an intervention rather than a therapeutic role, and thus can only make the intestinal microbial composition of the UC mice more similar to that of the BC mice. However, the species abundance of *Clostridia*, *Eubacterium*, and *Parabacteroides* in BLT mice was higher than in EM mice, indicating that BLT can alleviate obesity (Figure 10).

**FIGURE 11:**
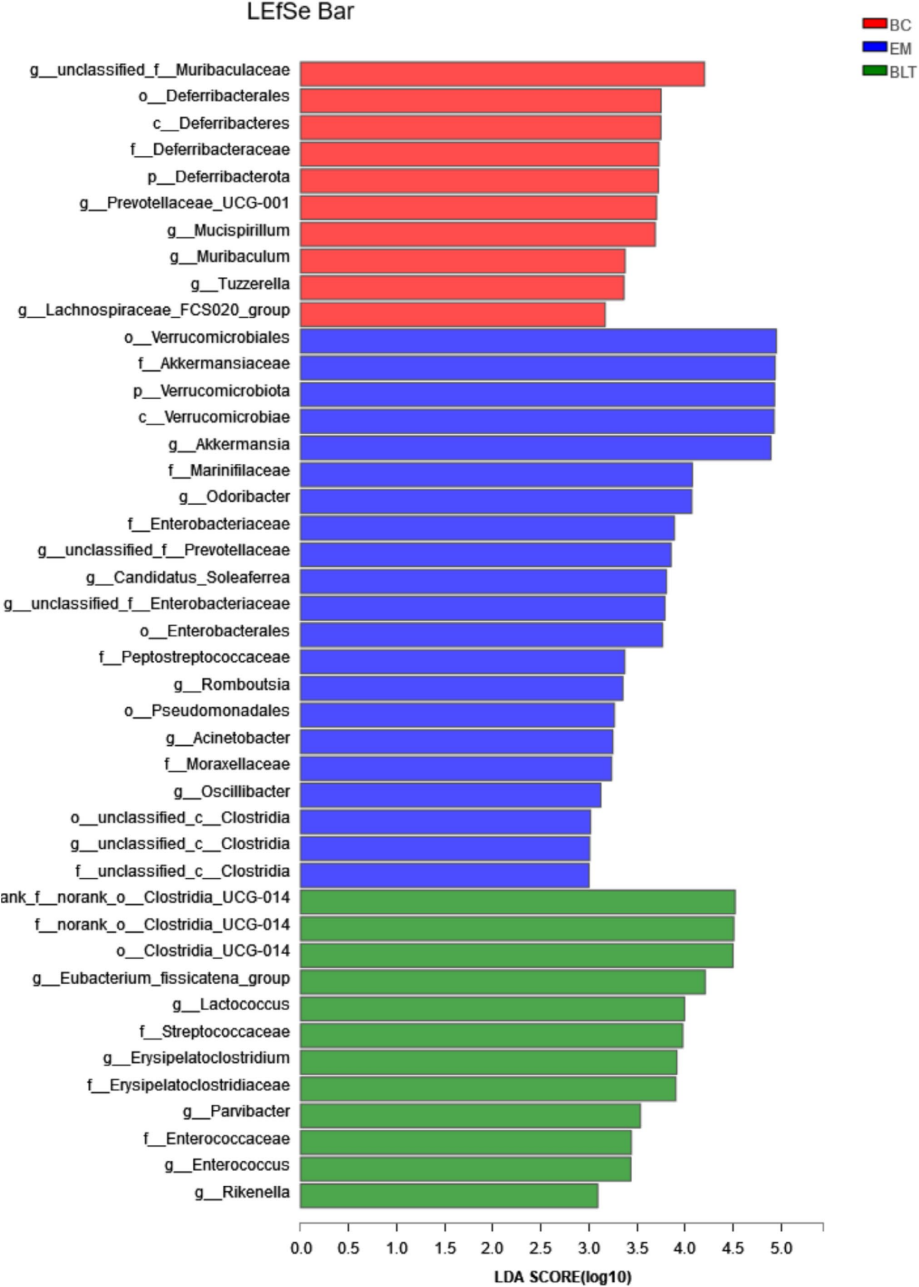
Histogram of LEfSe analysis of differential genus level among mouse gut microbiota groups.

The LEfSe linear analysis was used to estimate the influence of the abundance of each species on the differential effect (Figure 11). Compared with BC, the gut microbiota of mice with UC induced by DSS decreased the abundance of 17 genera and increased the abundance of 12 genera. Compared with the EM, the intestinal composition of mice with UC was significantly changed in the BLT intervention group, and the abundance of six genera was increased. Notably, the decreased abundance of 17 genera due to UC was not improved by the intervention of BLT.

**FIGURE 12:**
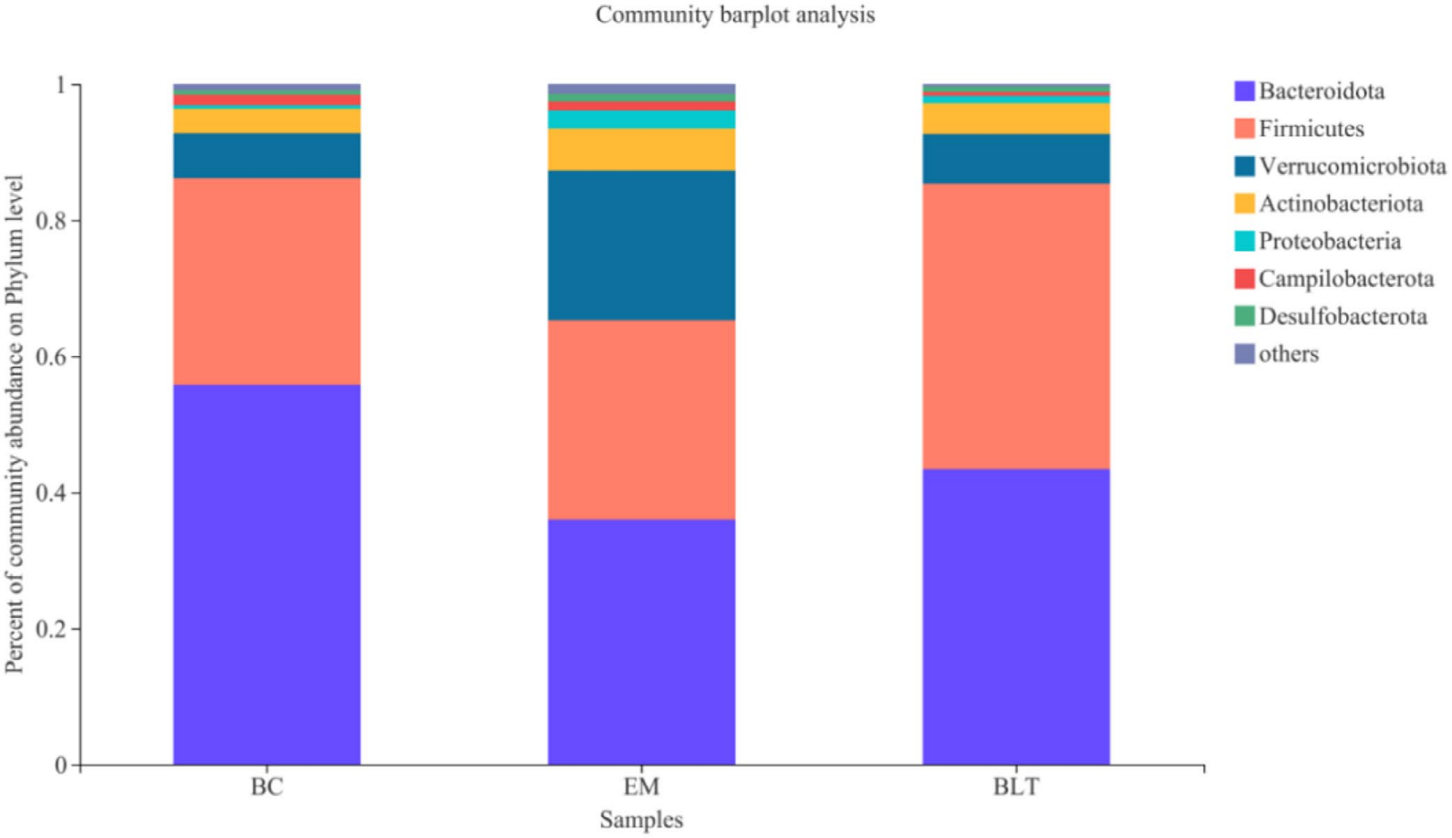
The structure of gut microbiota at the mouse phylum level.

As shown in Figure 12, the composition of mice gut bacteria is composed of five main phyla at the phylum level: including *Bacteroidota*, *Firmicutes*, *Verrucomicrobiota*, *Actinobacteriota*, and Proteobacteria. Compared with the BC, the abundance of *Bacteroidota* and *Firmicutes* decreased in the EM, while the abundance of *Verrucomicrobiota* and *Actinobacteriota* increased. Compared with the EM, the abundance of *Bacteroidota* and *Firmicutes* increased in the BLT intervention group, while the abundance of *Verrucomicrobiota* and *Actinobacteriota* decreased. These results indicated that UC induced by DSS could decrease the abundance of *Bacteroidota* and *Firmicutes* in the intestinal intestine of mice, and the abundance of these two *Bacteroidota* and *Firmicutes* could recover after the intervention of BLT.

## 4 Discussion

The inflammatory response induced by intestinal microorganisms and exogenous antigens is closely related to the development of UC (Paul and David 2007), and targeted therapy using proinflammatory factors TNF‐α, IL‐1β, and IL‐6 is commonly used in the clinical treatment of UC, which can effectively reduce intestinal damage caused by the inflammatory response (Rutgeerts et al. 2005). UC is a chronic gastrointestinal disease. Our previous studies have demonstrated that barley glucan is effective in treating the symptoms of UC mice (Chen et al. 2021). However, the intrinsic regulatory mechanisms of HB whole grain, HB bran, and peeled HB for reducing DSS‐induced UC mice have not been defined. So, the present studies investigate the preventive intervention treatment of DSS‐induced UC mice with different HB parts and then assess the effectiveness of their function separately. It should be noted that Zhou et al. (2019) modeled 6‐week‐old wild‐type C57BL/6 male mice in a 4% DSS solution and found a decrease in body weight from the fourth day, and the DAI score increased at the same time. However, in the present study, 8‐week‐old C57BL/6 male mice were modeled using a 2% (W/V) DSS solution, and no mice died throughout the study, with a tendency for mice to lose body weight from the third day onward, and diarrhea as well as blood in the stools from the fifth to the seventh day. This result is slightly different from that of Zhou et al. It is presumed to be related to the differences in the sex of the mice, the weekly age, and the concentration of DSS selected for this experiment from the literature.

In this study, mice in the UC model group that was induced by 2% DSS showed a sharp decrease in body weight, a significantly increased DAI, a significantly shorter colon length, and a change in fecal pattern from loose and soft to unformed to blood in the stool. However, the BLT (20%) was able to effectively treat the aforementioned symptoms and restore the mice to normal. Studies have shown that the generation of oxidative stress is a key factor in colonic injury, and infection causes oxidative stress and produces inflammatory mediators, which results in an inflammatory response (Batista et al. 2020; Marafini et al. 2019; Rapa et al. 2020). An analysis of the effects of oxidative stress levels in mice reveals that peeled HB reduces the decrease in oxidative stress capacity in mice caused by ulcerative colitis by inhibiting the decrease in GSH‐Px and SOD content, and GSH‐Px may protect against colonic injury (Santiago et al. 2015; Cui et al. 2019). Colonic macrophages are activated during the disease process to promote the secretion of IL‐1β and induce inflammatory damage (Luo et al. 2019). Analysis of the serum levels of inflammatory factors in all groups of mice reveals that intervention treatment with BLT can reduce the levels of IL‐1β in the serum of mice with UC and bring back the levels of IL‐10 in the serum of mice, suggesting that peeled HB can exert anti‐inflammatory effects. The findings presented above are comparable to Peng et al. (2019). Moreover, the increased IL‐10 production dramatically reduced inflammation in mice with UC brought on by DSS. In conclusion, BLT has a significant alleviating effect on the disease manifestation in mice with UC. In mice, BLT had the effect of raising serum anti‐inflammatory factor and decreasing serum proinflammatory factor, which reduced the organism's inflammatory response, and it also has the potential to increase GSH‐Px levels and SOD activity, hence enhancing the UC‐related oxidative stress. Therefore, a thorough investigation into the alleviation of intestinal injury in mice and the impact of BLT on the transcriptional levels of genes connected to colonic tissue followed.

Histopathological studies showed that the disruption of crypt structures, loss of epithelial cells, and inflammatory cell infiltration were significantly reduced in the colonic tissues of experimental mice treated with BLT intervention, indicating that the genes dysregulated in ulcerative colitis are mainly focused on the immune process (Dent et al. 2014) and that the over‐recruitment of activated neutrophils in the intestine causes inflammation (Fournier and Parkos 2012). In brief, interventional treatment with BLT resulted in a reduction in neutrophil infiltration and alleviated ulcerative damage in the colon in mice, which is consistent with the previously measured finding that BLT significantly reduced serum proinflammatory factors.

The body's inflammatory response has been found to be significantly influenced by the overproduction of proinflammatory factors and the loss of anti‐inflammatory factors (Motaghi et al. 2016). Excessive levels of IL‐1β, a closely related inflammatory factor produced by tissues' macrophages and peripheral blood monocytes, also cause pathological reactions in the body. TNF‐α is a key contributor to intestinal mucosal damage because it prevents intestinal epithelial cells from producing and expressing genes that prevent apoptosis. This increases the body's inflammatory response and further damages the intestinal mucosal barrier (Nenci et al. 2007). The intestinal environment will become unstable due to the expression of IL‐6, another important proinflammatory factor in UC, which can also directly activate the intestinal STAT3 pathway and exacerbate the inflammatory response in UC by impairing the secretory function of intestinal epithelial cells (Danese et al. 2019). The findings of this study indicate that BLT used as an intervention can reduce the levels of pro‐inflammatory factors in UC mice and control the expression level of transcription factors in UC‐related pathways, potentially easing the symptoms of UC.

Numerous clinical and animal experiments have shown that gut microbiota has become a new target for drug treatment of various diseases, and dysregulation of gut microbiota is closely associated with nonspecific intestinal inflammatory diseases (Anaïs et al. 2019; Yilmaz et al. 2019). Studies have shown that DSS can worsen intestinal mucositis by altering gut microbiota (Hou et al. 2021). There isn't any concrete proof that peeled HB can reduce DSS‐induced UC by altering the gut microbiota and enhancing the environment for that flora. The present study conducting the gut microbiota of mice given BLT preventive intervention was analyzed in order to determine whether the gut microbiota is involved in the modulation of DSS‐induced UC in mice. The analysis's findings demonstrated that the DSS‐induced loss of the intestinal microbiota in mice was reversed by the preventive intervention of BLT, which altered the abundance of the intestinal microbiota in those mice. Additionally, the intervention treatment of BLT upregulated the abundance of *Bacteroidota* and *Firmicutes* in mice. Studies have found that low abundance of *Firmicutes* increases intestinal susceptibility to inflammation (Jang et al. 2019; Rühlemann et al. 2019). The above results suggest that BLT can perform anti‐inflammatory effects by improving the balance of gut microbiota.

## 5 Conclusion

The results of this study showed that supplementation with a 20% dose of peeled HB restored body weight, DAI, colon length, IL‐1β and IL‐10 levels, liver GSH‐Px content, and SOD activity to normal levels in mice compared to UC mice. Moreover, the damage caused by UC to the mice's colon was significantly reduced, and the relative expression levels of IL‐1β, IL‐6, and TNF‐α were all significantly downregulated. Additionally, it increased the abundance of *Bacteroidota* and *Firmicutes*, improving the balance of gut microbiota. In conclusion, low‐dose peeled HB has great potential for the prevention of UC and related chronic diseases. This study will provide new insights into the prevention of UC. HB can be further developed into a sustainable crop to enhance human health.

## Author Contributions

**Huawei Liu:** conceptualization (equal), formal analysis (equal), resources (equal), writing – original draft (equal). **Wen Zhao:** conceptualization (equal), data curation (equal), resources (equal), writing – original draft (equal). **Hongzhou Chen:** conceptualization (equal), investigation (equal), validation (equal). **Hongya Wu:** formal analysis (equal), software (equal). **Xiangfei Li:** investigation (equal), validation (equal). **Anxiang Su:** investigation (equal), supervision (equal), writing – review and editing (equal). **Yingjian Lu:** funding acquisition (equal), supervision (equal), validation (equal), writing – review and editing (equal).

## Conflicts of Interest

The authors declare no conflicts of interest.

## Data Availability

The data that support the findings of this study are available on request from the corresponding author. The data are not publicly available due to privacy or ethical restrictions.
